# Complications in a Patient With Multiple Myeloma Mimicking Bronchopneumonia

**DOI:** 10.7759/cureus.99897

**Published:** 2025-12-22

**Authors:** Aruna Gowda, Pranathi Guruswamy, Aadithya Shyllesh H, TC Nagesh Kumar

**Affiliations:** 1 Internal Medicine, Ramaiah University of Applied Sciences, Bengaluru, IND

**Keywords:** ards, bronchopneumonia, lytic bone lesion, mods, monoclonal gammopathy, multiple myeloma, pneumonia

## Abstract

An elderly gentleman with a history of ischemic heart disease was admitted with respiratory distress. On evaluation, he exhibited renal dysfunction and elevated levels of total protein and globulin. The following day, the patient developed altered sensorium; further evaluation identified hypercalcemia, leading to a provisional diagnosis of hypercalcemic encephalopathy. A non-contrast CT scan of the brain revealed multiple lytic lesions in the skull, which were suspected to be a forerunning indicator for a possible multiple myeloma (MM). Bone marrow studies and protein electrophoresis further confirmed the same. A diagnosis of MM in the garb of an unsuspecting pneumonia was evident. Our patient was promptly initiated on chemotherapy alongside supportive therapy. However, he succumbed to illness following prolonged ICU admission, complicated by acute respiratory distress syndrome and multi-organ dysfunction. Hence, this case served as a reminder to consider the various agencies of pulmonary worsening in a patient with MM.

## Introduction

Multiple myeloma (MM) is a malignant monoclonal gammopathy that denotes the aberrant proliferation of malignant plasma cells producing monoclonal immunoglobulin. These myeloma cell clones can infiltrate the bone marrow, resulting in marrow failure, and produce pathological fractures with osteolytic lesions and abnormal protein (M component) production, resulting in kidney injury, secondary amyloidosis, and hyperviscosity syndrome. Proliferating myeloma cells in the bone marrow result in cytopenias, including leukopenia, that precipitate immune deficiency, making patients susceptible to infections.

Mechanisms such as hypogammaglobulinemia, immunoparesis, or impaired lymphocyte function in active myelomas predispose these patients to infectious complications [1,2]. Furthermore, the initiation of chemotherapeutic drugs and immunomodulators following the diagnosis of MM also increases the susceptibility to infections. Pulmonary parenchymal involvement by proliferating clonal plasma cells is very rare. However, extramedullary plasma cell neoplasms have been reported in the lungs [3]. Distinguishing the same from a pulmonary infection can be challenging.

We hereby report the case of a rare manifestation of MM where the patient initially came with an unsuspecting lower respiratory tract infection, which, on further investigation, prompted the incidental diagnosis of MM. This case was unique in its uncommon presentation of MM mimicking bronchopneumonia thereby emphasizing the importance of diagnostic vigilance. 

## Case presentation

**Figure 1:**
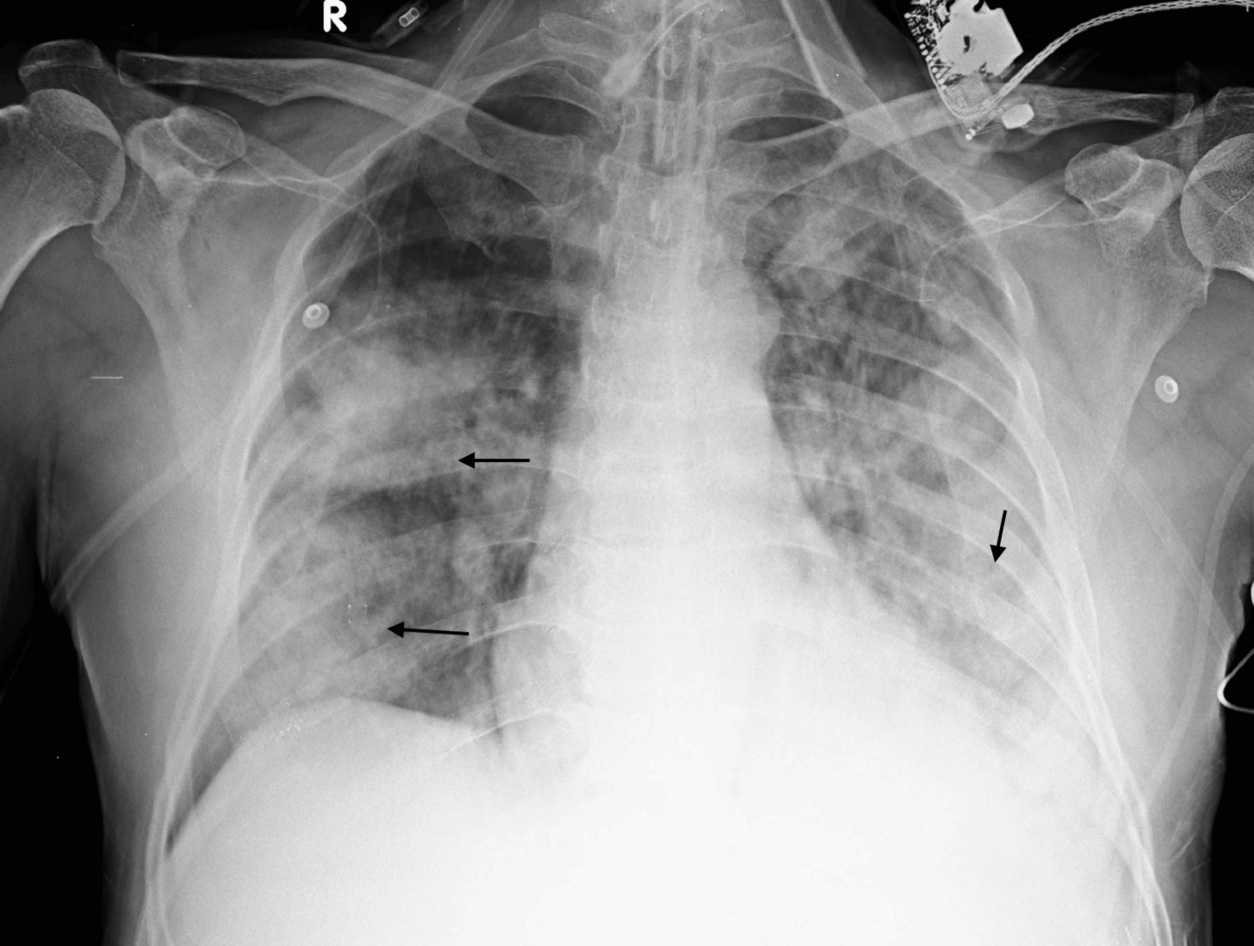
Chest X-ray on presentation showing consolidations with air bronchograms in bilateral mid and lower zones of the lungs, along with fluffy non-homogeneous opacities consistent with pneumonia and early ARDS ARDS: Acute respiratory distress syndrome

A 67-year-old man with a history of ischemic heart disease presented with a three-day history of cough and a one-day history of breathlessness. On admission, he exhibited tachypnea, tachycardia, and hypoxia, with intact consciousness and orientation. Bilateral crepitations were noted on chest examination, and a chest X-ray revealed non-homogeneous opacities in the bilateral mid and lower lung zones (Figure 1). High-resolution CT imaging of the lungs was pursued, which was suggestive of consolidations with air bronchograms in the left lower lobe and right middle and lower lobes of the lungs, with surrounding ground glass opacities. The possibility of a non-cardiogenic pulmonary edema tending towards acute respiratory distress syndrome (ARDS), secondary to an infective etiology, was considered. Hence, the patient was promptly admitted to the ICU and commenced on oxygen therapy and broad-spectrum dual antibiotics comprising oral azithromycin and IV piperacillin-tazobactam. Initial investigations indicated mild anemia (Hb - 12.5), thrombocytopenia (92,000), and neutrophilic leukocytosis (total leukocyte count - 15,750/mm³) (Table 1). Renal parameters showed an elevated creatinine of 3.01 mg/dL with metabolic acidosis. In line with an infective workup, the patient was found to have raised CRP (26 mg/L) and negative procalcitonin of 0.05 ng/ml. The sputum sample grew normal upper respiratory flora on culture. Furthermore, paired blood cultures also yielded no significant growth, thereby making the initial diagnostic impression of bronchopneumonia seem dubious.

**Table 1:** Laboratory parameters of the patient on initial presentation Alb, albumin; ALT, alanine aminotransferase; ALP, alkaline phosphatase; APTT, activated partial thromboplastin time; AST, aspartate aminotransferase; B, basophils; BUN, blood urea nitrogen; CXR, chest X-ray; DB, direct bilirubin; DLC, differential leukocyte count; EF, ejection fraction; EEG, electroencephalogram; E, eosinophils; ESR, erythrocyte sedimentation rate; Free T₃, free triiodothyronine; Free T₄, free thyroxine; GGT, gamma-glutamyl transferase; GRBS, gross random blood sugar; Hb, hemoglobin; HCO₃⁻, bicarbonate; IVC, inferior vena cava; INR, international normalized ratio; L, lymphocytes; LV, left ventricle; M, monocytes; N, neutrophils; PASP, pulmonary artery systolic pressure; PCV, packed cell volume; PLT, platelets; PT, prothrombin time; QT, QT interval; QTc, corrected QT interval; RWMA, regional wall motion abnormality; TB, total bilirubin; TLC, total leukocyte count; TP, total protein; Trop-T, troponin T; TSH, thyroid-stimulating hormone

Parameters	Values	Reference Range
Complete blood count	Hb- 12.5 g/dL	13-17 g/dL
	TC- 15750 cells/cumm	4000 – 11000 cells/cumm
	PLT- 1.80 lakhs/cumm	1.5 – 4.0 lakhs/cumm
Differential count	N- 64.8%	40 – 80 %
	L- 21.3%	20 – 40 %
	E- 1.0%	1 - 6 %
	M- 12.7%	2 – 10 %
	B- 0.2%	0 – 1 %
ESR	70 mm/hr	0 – 20 mm/hr
Peripheral smear	Normocytic normochromic blood picture	
Coagulation profile	PT- 21.2 secs	11.51 – 15. 51 secs
	aPTT- 26.7 secs	25.46 – 29.46 secs
	INR- 1.60	0.8 – 1.2
Renal profile		
Blood urea nitrogen	69.20 mg/dL	7 – 20 mg/dL
Creatinine	3.01 mg/dL	0.6 – 1.2 mg/dL
Uric acid	19.5 mg/dL	3.5 – 7.2 mg/dL
Electrolytes	S. Sodium- 141 mmol/L	136 – 145 mmol/L
	S. Potassium- 3.6 mmol/L	3.5 – 5.1 mmol/L
	S. Chloride- 96 mmol/L	96 – 104 mmol/L
	S. calcium- 18.5 mg/dL	8.5 – 10.5 mg/dL
Arterial blood gases	pH- 6.90	7.34 – 7.45
	pO2- 56 mmHg	75 – 100 mmHg
	pCO2- 43 mmHg	35 – 45 mmHg
	HCO3- 16 mmol/L	22 – 26 mmol/L
	Lac- 2.40 mmol/L	0.36 – 0.75 mmol/L
	SO2- 63%	95- 98%
Liver profile		
Total bilirubin	1.88 mg/dL	0.1 – 1.2 mg/dL
Direct bilirubin	0.84 mg/dL	0.0 – 0.3 mg/dL
Alanine transaminase	95 U/L	19 – 48 U/L
Aspartate transaminase	32 U/L	19 – 48 U/L
Alkaline phosphatase	95 U/L	55 – 119 U/L
Gamma glutamyl transferase	95 U/L	0 – 55 U/L
Total protein	10.1 g/dL	4.4 – 7.6 g/dL
Albumin	3.6 g/dL	3.20 – 4. 60 g/dL
A/G ratio	0.6	1.0 – 2.5
Endocrine Profile		
	RBS- 115 mg/dL, HBA1c – 6.2%	70 – 110 mg/dL
	TSH – 1.34 microU/mL	0.5 – 8.9
HIV/HBsAg/Anti-HCV	Non- reactive	
Beta 2 microglobulin	35.37 mg/L	1.5 – 3 mg/L
Serum protein electrophoresis		
Free kappa light chains	25.1 mg/L	3.3 – 19.4 mg/L
Free lambda light chains	109 mg/L	5.7 – 26.3 mg/L
Bone marrow biopsy	38% clonal plasma cells	

The lack of clinical findings such as pedal edema, raised JVP, and a normal NT-proBNP with normal baseline ECG made the possibility of cardiogenic pulmonary edema as a cause for dyspnea unlikely. With acute onset of respiratory distress, bilateral lung opacities on chest imaging and no evidence suggestive of cardiogenic pulmonary edema, a diagnosis of ARDS was forthcoming on presentation itself. However, the precipitating factor for ARDS-like clinical picture was yet to be ascertained.

**Figure 2:**
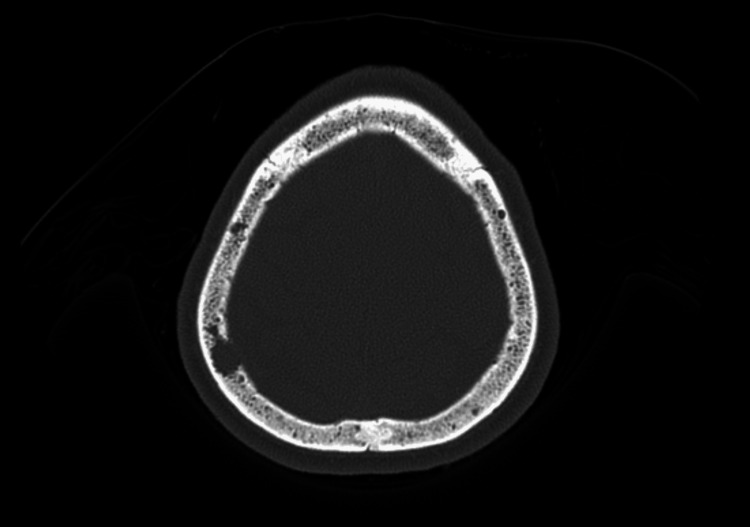
Cross-sectional view of CT skull with multiple lytic bony lesions

A close observation of the patient's liver function parameters revealed elevated total protein levels and albumin-globulin reversal, prompting a quest in line with MM. On the second day of admission, the patient developed new-onset altered sensorium, prompting an emergency CT scan of the brain; the scan revealed multiple lytic lesions in the skull (Figure 2), further strengthening our suspicion of MM. A bone marrow biopsy was performed, which demonstrated 38% clonal plasma cells and raised blood free lambda light chains of 109 mg/L (normal range: 5.71 - 26.3). Serum protein electrophoresis identified a marked monoclonal gamma globulin spike suggestive of monoclonal gammopathy, with an M-protein concentration of 4.1 g/dL. Additionally, beta 2 macroglobulin was elevated at 35.37 mg/L. Based on these findings, the patient was diagnosed with ISS Stage II MM in concurrence with the medical oncology team. The patient was initiated on chemotherapy with bortezomib, dexamethasone, and cyclophosphamide by day 4 of hospital admission. The need for bronchoscopy and bronchoalveolar lavage (BAL) for demonstrating monoclonal plasma cells was discussed with pulmonology and critical care teams; however, it could not be performed due to limitations raised by the family members. With the backdrop of MM, the initial pulmonary findings suggestive of ARDS could be attributed to the underlying disease process itself, although conclusive evidence of the same by means of lung biopsy, BAL fluid analysis or CT findings of metastatic calcifications was not forthcoming.

**Figure 3:**
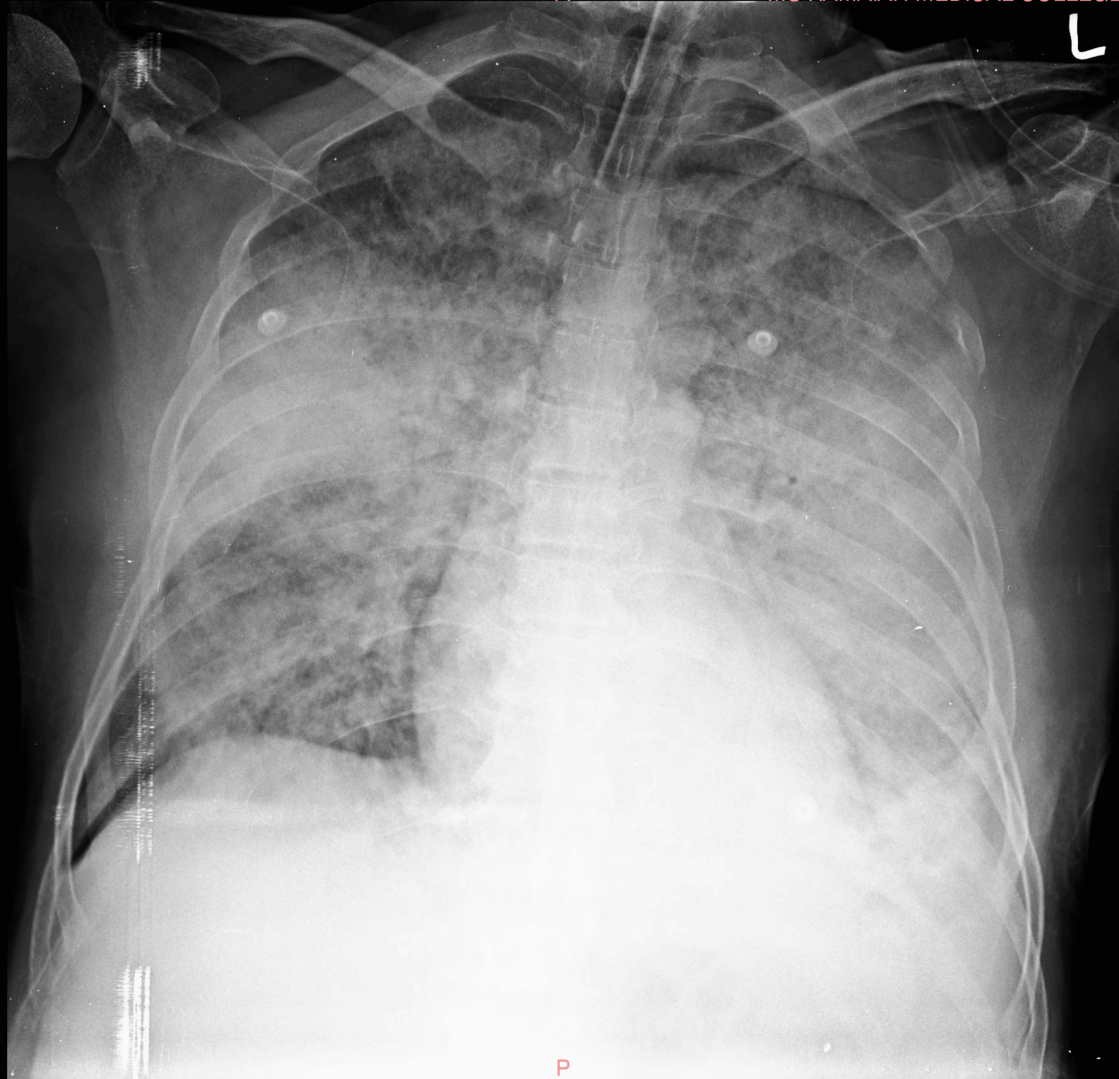
Chest X-ray demonstrating a worsening non-cardiogenic pulmonary edema consistent with ARDS ARDS: Acute respiratory distress syndrome

Soon after, on the fifth day of ICU admission, the patient developed worsening tachypnoea with worsening respiratory status. A repeat chest imaging showed non-homogenous opacities consistent with a worsening acute respiratory distress syndrome (Figure 3). In response, he was intubated and initiated on mechanical ventilation. Furthermore, a hypercalcemia of 18.5 mg/dL and persistent metabolic acidosis necessitated hemodialysis. A repeat infection profile comprising total leucocyte counts, procalcitonin, blood and urine cultures ruled out the possibility of a superinfection. However, broad-spectrum prophylactic antibiotics were continued. In the following week of ICU stay, he required multiple sessions of renal replacement therapy in view of progressive deterioration of renal function with anuria and persistent metabolic acidosis. He was continued on ventilatory support alongside chemotherapy. Despite prompt diagnosis of monoclonal gammopathy and initiation of chemotherapy alongside appropriate resuscitative measures, the patient succumbed to death on the 10th day of ICU admission with multiorgan dysfunction syndrome.

## Discussion

MM is a proliferative disorder of clonal plasma cells marked by the abnormal overproduction of monoclonal immunoglobulins. Left unchecked, this excessive plasma cell growth can result in targeted damage to specific organs. Typically, clinical signs manifest as hypercalcemia, renal dysfunction, anemia, or bone pain accompanied by bony lytic lesions [4].

The exact etiology of MM is unknown. However, frequent alterations and translocations in the promoter genes, especially chromosome 14, are commonly found in MM and likely play a role in disease development [5]. In addition, other oncogenes such as NRAS, KRAS, and BRAF may participate in plasma cell proliferation [5]. Other factors contributing to disease occurrence include obesity, alcohol consumption, environmental causes such as insecticides, organic solvents, Agent Orange, and radiation exposure [6,7]. Regardless of the underlying molecular cause, an excess of monoclonal immunoglobulins can lead to hyper viscosity, platelet dysfunction, and damage to renal tubules. This can result in neurological impairments, bleeding disorders, and renal failure, respectively. Additionally, bone marrow infiltration by the proliferating plasma cell clone typically presents as anemia, thrombocytopenia, and leukopenia.

The original CRAB criteria for the diagnosis of MM includes hypercalcemia (serum calcium >0.25 mmol/L higher than the upper limit of normal), renal insufficiency; creatinine clearance <40 mL per minute or serum creatinine >2 mg/dL, anemia with hemoglobin value >20 g/L below the lowest limit of normal or hemoglobin <100 g/L and one or more osteolytic lesions on skeletal radiography, CT, or PET/ CT. However, newer diagnostic criteria for MM include the following myeloma-defining events: Bone marrow involvement 60% or greater clonal plasma cells, serum involved/uninvolved free light chain ratio of 100 or greater, and focal lesions on MRI, more than one focal lesion that is at least 5 mm or greater in size.

The preferred treatment option is autologous stem cell transplantation. However, for patients who are not eligible for this procedure, initial therapy typically consists of chemotherapy with a regimen based on bortezomib. With newer chemotherapeutic drugs, stem cell transplantation, and recent advances, the disease largely remains incurable; however, it can be brought into remission with meticulous treatment and follow-up. 

Pulmonary involvement in MM resulting from extramedullary dissemination of monoclonal plasma cells to the lungs is well documented in medical literature [8]. Such involvement may present with respiratory distress and can easily be misinterpreted as a benign condition such as pneumonia. In addition, metastatic calcification within the pulmonary vasculature in MM can manifest as ARDS, further complicating the clinical presentation. This lung calcification can be attributed to worsening renal parameters including hyperkalemia and hypercalcemia. Respiratory distress can also be attributed to flail chest as a result of multiple osteolytic lesions in the ribs, leading to destabilization of the ribcage.

The principal challenge during this patient’s clinical course was the rapid and aggressive progression of the underlying disease despite the earliest possible initiation of chemotherapy. Additional factors contributing to mortality included renal failure with anuria, severe and persistent metabolic acidosis with hypercalcemia, and ongoing respiratory distress refractory to supportive measures such as multiple sessions of hemodialysis and mechanical ventilation.

This case underscores an atypical pulmonary manifestation of MM, where ARDS at presentation closely mimicked bronchopneumonia, leading to an initial misdirection in clinical assessment and management. Although the diagnosis was eventually established, timely therapeutic intervention could not be achieved, and the patient ultimately succumbed to MM with progressive complications, including ARDS and renal failure. The key learning point from this case is that a seemingly common presentation such as bronchopneumonia may obscure an underlying malignancy like MM. Furthermore, the clinical course illustrates that even with prompt recognition, aggressive and rapidly progressive disease may still result in poor outcomes despite appropriate management.

## Conclusions

In conclusion, pulmonary manifestations in patients with MM can present in a variety of ways, as discussed above. Clinicians must remain vigilant to these often-misleading presentations. As seen in this case, it becomes pertinent to anticipate and prepare for the severity and aggressive nature of associated complications. This case further reinstates the importance of timely recognition and initiation of chemotherapy in MM to avert its life-threatening complications.
